# Surgery for retroperitoneal relapse in the setting of a prior retroperitoneal lymph node dissection for germ cell tumor

**DOI:** 10.4103/0970-1591.60452

**Published:** 2010

**Authors:** Geoffrey T. Gotto, Brett S. Carver, Pramod Sogani, Joel Sheinfeld

**Affiliations:** Department of Urology, Memorial Sloan-Kettering Cancer Center,Weill College of Medicine,New York, USA; 1 Department of Urology, Weill College of Medicine, New York, USA

**Keywords:** Recurrence, relapse, reoperative, retroperitoneal lymph node dissection, testicular cancer

## Abstract

Recognition of the therapeutic role of retroperitoneal lymph node dissection (RPLND) in the setting of testicular germ cell tumors (GCTs) is of utmost importance. Although the histologic findings of RPLND provide diagnostic and prognostic information, the adequacy of initial RPLND is an independent predictor of clinical outcome. Despite the advent of effective cisplatin-based chemotherapy for testicular GCTs, patients who have undergone suboptimal surgery at the time of initial RPLND are compromised. Despite the initial enthusiasm surrounding anatomic mapping studies, the use of modified RPLND templates has the potential to leave a significant number of patients with unresected retroperitoneal disease. Teratomatous elements are particularly common. Patients with retroperitoneal relapse following initial RPLND should be treated with reoperative RPLND and chemotherapy and can expect long term survival rates nearing 70% when treated in tertiary centers by experienced surgeons.

## INTRODUCTION

Testicular cancer is generally regarded as highly curable even in the setting of advanced disease. This is in large part due to the advent of effective cisplatin-based chemotherapy.[1] Retroperitoneal lymph node dissection (RPLND) remains an integral component of the management of patients with testicular germ cell tumors (GCTs) in both the primary and post-chemotherapy settings. When combined with systemic chemotherapy, overall survival rates of greater than 90% can be expected.[2]

RPLND should always be pursued with therapeutic intent whether it is in the primary or post-chemotherapy setting. While pathologic findings following RPLND are important in the diagnosis and staging of patients with testicular GCTs, it cannot be overstated that this procedure can have a profound impact on clinical outcomes when properly performed.[3,4] When patients relapse in the retroperitoneum after an inadequately performed RPLND even aggressive cisplatin-based chemotherapy protocols will not result in cure in all men.[4–7] Every attempt should be made to resect all retroperitoneal disease at the time of initial RPLND and this goal may not be consistently achieved using restricted, modified templates described in the literature.

Patients who undergo an inadequate initial RPLND are at risk for relapse in the retroperitoneum.[7] There is a role for reoperative RPLND in these patients although oncologic outcomes are compromised when compared with patients who underwent initial RPLND with complete surgical resection.[4–7] Although the use of modified surgical templates has been shown to aid in preservation of antegrade ejaculation, similar results can be achieved with a bilateral nerve-sparring procedure without compromising relapse-free survival. The adequacy of the surgical resection at the time of initial RPLND is an independent predictor of relapse-free survival in both low-stage and advanced nonseminomatous germ cell tumor (NSGCT).[4,8]

This article will describe the patient population in which reoperative RPLND should be considered and will review sites of recurrence and histologic findings at the time of reoperative RPLND. The surgical morbidity and oncologic outcomes of reoperative RPLND will be addressed. Finally, this article includes a discussion on what puts patients at risk for retroperitoneal recurrence in the setting of a previous RPLND and how to avoid compromising relapse-free and cancer-specific survival at the time of initial RPLND.

## RETROPERITONEAL METASTASES IN TESTICULAR GCTS

Regardless of histologic subtype, testicular GCTs tend to spread through retroperitoneal lymphatics in a predictable fashion. This is felt to be the result of embryologic migration of the testis through the retroperitoneum where it acquires drainage from the lymphatics adjacent to the aorta and inferior vena cava.[9] It is uncommon to observe metastases to the lung and posterior mediastinum without concomitant retroperitoneal disease.[9,10] Choriocarcinoma does show a higher rate of hematogenous spread, resulting in distant metastases without apparent retroperitoneal involvement, but still tends to follow a similar pattern to the other subtypes the majority of the time.[10]

Right-sided testicular GCTs tend to spread first to the interaortocaval region followed by the precaval and preaortic nodes. In contrast, the primary metastatic sites for left-sided tumors are the paraaortic and preaortic nodes.[9,11–13] However, mapping studies have clearly established that multifocality and contralateral spread are more common with higher pathologic stage. This is particularly true for right-sided tumors.[10–13] Subsequent spread through the retroperitoneum is typically in a cranial direction although caudal spread to the iliac, pelvic, and inguinal nodes can occur in the setting of bulky retroperitoneal disease.[10–13]

## RETROPERITONEAL LYMPH NODE DISSECTION (RPLND)

The retroperitoneum is the initial, and often only, site of metastatic spread in up to 90% of patients with GCT.[10] It also represents the most common site for late relapse of both teratoma and viable GCT.[5,7,14] Despite improvements in radiographic imaging, up to 30% of patients who present initially with viable tumor in the retroperitoneum are clinically under staged.[10] Metastases to the brain, bone, and liver are typically late events and are typically seen only in association with bulky retroperitoneal disease.[9,15,16]

The indications for RPLND and the surgical template for dissection have changed significantly since its role was first established in 1948.[9] Bilateral suprahilar dissection was at one time the standard of practice.[9] With the advent of effective cisplatin-based chemotherapy, routine suprahilar dissection was abandoned due to its association with renovascular injury, pancreatic leak, and chylous ascites, which accounted for much of the surgical morbidity associated with RPLND.[9]

Retrograde ejaculation remained a significant risk even with bilateral infrahilar RPLND. The incidence of this complication is related to the extent of retroperitoneal dissection.[9,17] A number of modified side-specific templates have been proposed with the objective of minimizing damage to the contralateral paravertebral sympathetic ganglia, postganglionic sympathetic fibers, and hypogastric plexus.[9] A common feature of most of these templates is a limited dissection below the inferior mesenteric artery (IMA) on the contralateral side.[9,18,19] However, antegrade ejaculation can be maintained with the application of nerve-sparing techniques in which these structures are prospectively identified, dissected and preserved, in the setting of a full bilateral template dissection.[9,20,21]

## RETROPERITONEAL RECURRENCE AFTER RPLND

The issue of retroperitoneal relapse after RPLND is not commonly addressed in the literature. Its true incidence is likely underestimated due to

- widespread use of postoperative cisplatin-based therapy
- lack of standardized routine imaging following RPLND
- limited follow-up periods in published series

Postoperative cisplatin-based chemotherapy eliminates occult micrometastatic disease in a significant proportion of patients. Most protocols for follow-up after RPLND include plain chest radiographs and serum tumor markers but not routine imaging of the retroperitoneum. When a relapse is detected, the patient is often treated with chemotherapy without imaging of the retroperitoneum to document the precise site of relapse. For this reason, many reports in the literature fail to report the site of recurrence. Finally, retroperitoneal relapse in the setting of a prior RPLND is commonly a late event which is likely missed in many published series due to insufficient follow-up.[9,22–24] In patients with late relapse, the retroperitoneum is involved in 72% and 36% of patients present at least 10 years after an initial complete response.[7]

Relapse in the retroperitoneum in the setting of a prior RPLND results from suboptimal surgery in the vast majority of cases. Failure to completely resect retroperitoneal disease may be the result of technical errors or inappropriate modification of surgical templates.[23–25] Limited surgical volume and inexperience have been shown to compromise clinical outcomes in a variety of complex surgical procedures.[26–28] Given the relatively low incidence of testicular cancer, and declining use of primary RPLND, it is a challenge for many surgeons to maintain a high volume of cases. Even residency training programs struggle to maintain reasonable exposure to this procedure.

A review of operative log reports from 2000 through 2004 by the Accreditation Council for Graduate Medical Education Residency Review Committee for Urology revealed that the average number of RPLNDs performed by residents graduating in the United States in 2004 during their entire residency training was four. Roughly half of these residents acted as the primary surgeon in two or less RPLNDs and as first assistant in one or less.[29] The bottom tenth percentile were not involved in a single RPLND as primary surgeon or first assistant.[29]

## MODIFIED SURGICAL TEMPLATES FOR RPLND

Modified templates for RPLND are based on anatomic mapping studies by Ray and Whitmore, Donohue and Weissbach.[11–13] The findings of these studies suggest that certain regions are at lower risk for metastases according to the side of testicular involvement.[11–13] The basic premise for modified templates is resection of the ipsilateral lymph nodes between the renal vessels and the bifurcation of the common iliac artery, with contralateral dissection limited or omitted. In the case of left-sided primary tumors, interaortocaval dissection is variably performed depending on the template.[9]

The limitations of modified templates were first addressed by Jewett and Torbey in 1988.[30] The mapping studies on which modified templates are based have been shown to underestimate the true extent of retroperitoneal involvement. There are several reasons for this. First, true rates of recurrence outside the modified template cannot be assessed without adequate follow-up. Node-negative patients were followed for a median of only 22 months in the Weissbach study during which time three retroperitoneal relapses were noted.[13] The studies by Ray and Whitmore and Donohue reported no follow-up at all.[11,12] Second, despite being the only mapping study with any patient follow-up, the Weissbach study randomized patients with pathologic stage IIB disease to either two or four cycles of cisplatin-based chemotherapy which precludes accurate assessment of retroperitoneal relapse.[13] Third, variability in surgical technique and adherence to the modified template likely influenced the results of the Weissbach study which included patients from 50 surgeons in 46 different centers.[13] Finally, renal and renovascular anatomic variants, including variable insertion and branching of the right gonadal vein, may influence lymphatic drainage of the testis resulting in metastatic deposits outside a modified template.

Two recent publications from MSKCC examined rates of extra-template disease in patients undergoing either primary or post-chemotherapy RPLND.[18,31] Eggener *et al*. evaluated 191 clinical stage I-IIA NSGCT patients who underwent full bilateral primary RPLND and were found to be node-positive. The incidence of node-positivity outside of five commonly referenced modified templates ranged from 3%-23%.[18] Carver *et al*. evaluated 269 patients with viable GCT or teratoma at the time of post-chemotherapy RPLND for advanced NSGCT and found that, depending on the limits of the modified template, 7-32% had viable extra-template disease.[31] Neither study found a difference in the histologic distribution of extra-template disease compared to in-template disease.[31]

Eggener *et al*. found that in patients with metastatic extra-template disease, the most commonly involved sites outside of modified right-sided templates were the pre-aortic (100%) and para-aortic (68-100%) regions.[18] Interestingly, a study by Leibovich *et al*, in 2002 identified these two regions as the sole sites of positive nodes in 28 of 607 patients (5%) with right-sided NSGCT who underwent RPLND at Indiana University.[32] Eggener *et al*. identified the inter-aortocaval region as the most commonly involved site outside of modified left-sided templates with positive nodes found at this site in 88% of patients with extra-template disease.[18] Indeed, masses in the left para-aortic and/or hilar regions accounted for 50-53% of retroperitoneal masses resected at the time of reoperative RPLND in series from MSKCC and from Cespedes and Peretsman.[4,22] Another reason for inadequate resection at this site is difficulty in achieving adequate exposure which requires pancreatic mobilization and meticulous dissection of the left renal vessels.[4,9]

## CLINICAL IMPLICATIONS OF AN UNCONTROLLED RETROPERITONEUM

**Figure 1:**
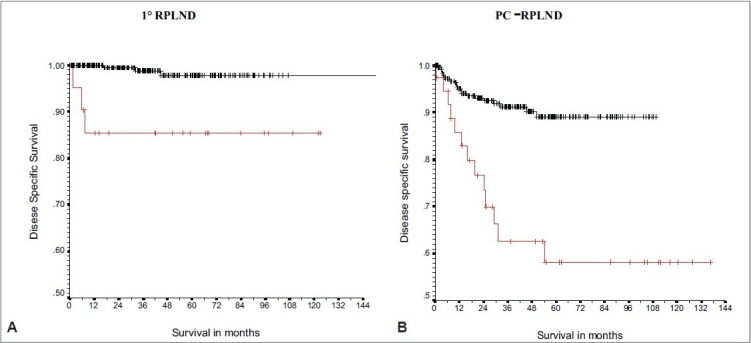
Adverse Impact of Redo-RPLND. Disease-specific survival for patients undergoing reoperative retroperitoneal surgery following primary RPLND (A) and PC-RPLND (B).[4]

RPLND must be performed with therapeutic intent. Even in the setting of primary RPLND, those patients with retroperitoneal relapse have compromised survival. As depicted in Figure 1A, 5-year cancer-specific survival (CSS) dropped from 99% to 86% for 22 patients who underwent redo-RPLND at MSKCC.[4] This decrease in survival occurred despite the fact that 20 of the 22 patients (90%) received two to four cycles of cisplatin-based chemotherapy as adjunctive therapy following primary RPLND (one patient) or because of incomplete resection (nine patients) or clinical relapse (10 patients) prior to undergoing reoperative RPLND. Cisplatin-based chemotherapy will not reliably compensate for an incomplete RPLND performed without therapeutic intent. Completeness of resection is also an independent predictor of CSS in the setting of late retroperitoneal relapse. A second study by Sharp *et al*. from MSKCC found that five-year CSS was 79% vs. 36% for patients with and without a complete resection performed at the time of late retroperitoneal relapse.[7]

Completeness of resection at the time of reoperative retroperitoneal surgery has been also been shown to be a significant and independent factor in the prognosis of patients with residual retroperitoneal disease.[4,7,8] Rates of disease progression have been shown to increase as much as 4-fold in patients with incompletely resected residual metastases.[7,33–35]

Two independent publications from Indiana University and MSKCC evaluated relapse and survival rates in patients undergoing either primary PC-RPLND or reoperative PCRPLND.[4,8] Donohue *et al*. report relapse rates for primary PC-RPLND versus reoperative PC-RPLND of 20.6% and 51.6%, respectively. Survival rates dropped from 84% in the primary group to 55% in the reoperative group.[8] Similarly, McKiernan *et al*. from MSKCC reported a drop in CSS from 90 to 56%.[4] These results are shown in Figure 1B.

The three most important prognostic factors for patients requiring reoperative surgery are serum tumor marker status at the time of reoperation, histologic findings at surgery, and completeness of resection.[4,7 36,37] In the study by McKiernan *et al*. patient survival at two years, post-reoperative RPLND was reported at only 52% for patients with elevated serum tumor markers at the time of surgery in contrast to those with normal markers who had survival rates approaching 80%.[4] With regards to histologic findings at the time of reoperative RPLND, there was significant variation in CSS for patients with fibrosis or resected teratoma (80%), viable GCT (44%), and teratoma with malignant transformation (20%).[4] The study by Sharp *et al*. from MSKCC found that in patients with late relapse specifically, a symptomatic presentation and multifocal disease were the only independent predictors of reduced CSS in a multivariable model, with hazard ratios of 4.9 and 3.0, respectively.[7]

The most common histologic finding at the time of reoperative retroperitoneal surgery following either primary RPLND or PC-RPLND is teratoma. Mc Kiernan reported that in patients with retroperitoneal recurrences following primary RPLND or PC-RPLND, teratoma was identified at the time of initial resection in 45% and 59% of patients, respectively.[4] Despite its benign histologic appearance, teratoma can recur locally and undergo malignant transformation. Therefore, complete resection is of all retroperitoneal teratoma is imperative. Late relapse from unresected teratoma has been reported in several series and the true incidence of this event is likely underreported because of inadequate follow-up.[9,23,38] Mass effects from growing teratoma may lead to invasion and/ or obstruction of surrounding structures.

Malignant transformation with sarcomatous and/or carcinomatous elements has been reported.[9] A study by Loehrer *et al*. reported late recurrence in 19% of patients with teratoma identified at the time of initial RPLND.[38] The majority of these relapses occurred within the retroperitoneum suggesting incomplete resection at the time of initial RPLND and malignant transformation was identified in a subset of patients.[38] Malignant transformation was responsible for two deaths following late retroperitoneal relapse of teratoma in a study by Holzik *et al*.[39] Survival for patients with teratoma with malignant transformation discovered at the time of reoperative RPLND is exceptionally poor at only 20%.[4]

## COMPLICATIONS OF REOPERATIVE RPLND

Achieving a complete resection at the time of reoperative RPLND is critical to avoid a repeat retroperitoneal recurrence and optimize oncologic outcomes including relapse-free and cancer-specific survival. Several groups have published on the morbidity associated with reoperative retroperitoneal surgery. Reoperative surgery is considered more challenging because of desmoplastic reaction and adhesions related to extravasation of blood and/or lymphatic fluid at the time of prior surgery and chemotherapy. This can result in difficulty with identification of planes and can lead, for example, to subadventitial vascular dissection and the need for vascular grafting.[40] Sacrificing adjacent organs (kidney, bowel, spleen) and/or graft replacement of a great vessel may be necessary.[9,35]

A small study published by Waples and Messing in 1993 reported on morbidity following reoperative retroperitoneal surgery in nine patients. Mean anesthetic time was 9.5 hours with a mean estimated blood loss of 6.3 liters. Five patients (56%) had significant perioperative complications including common bile duct injury, chylous ascites, and major vascular injury resulting in lower extremity amputation.[41]

More recently, in 2003, two separate publications reported on the experiences with reoperative RPLND at MSKCC and MD Anderson.[4,40] McKiernan reported an overall complication rate of 27% in 61 patients operated on at MSKCC.[4] The most common complications included lymphocele (4), ileus (3), wound infection (2), SBO (2), ureteral injury (2) and renal infarction (1).[4] There was one death due to pulmonary embolus.[4] Sexton *et al*. reported on 21 patients who underwent reoperative RPLND at M.D. Anderson. Intraoperative complications were reported in 29% of patients. Postoperative complications, including prolonged ileus and chylous ascites, were reported in 48%, and, again, there was a single death attributed to pulmonary embolus.[40] The morbidity of reoperative retroperitoneal surgery is minimized when performed in dedicated tertiary centers by experienced surgeons.[4,8,9,40]

## CONCLUSIONS

While RPLND does provide both diagnostic and prognostic information, it is first and foremost a therapeutic procedure. Failure to recognize this may lead to suboptimal surgery putting patients at risk for late relapse and compromised clinical outcomes. While reoperative surgery in combination with cisplatin-based chemotherapy can salvage nearly 70% of patients with retroperitoneal relapse after a suboptimal RPLND, inadequate initial resection of retroperitoneal metastases has been shown to be a an independent adverse prognostic variable in NSGCT. There is significant limitation to the anatomic mapping studies used to justify the use of modified surgical templates and, at present, no modification of the standard bilateral RPLND template will result in reliable resection of all retroperitoneal disease in either the primary or post-chemotherapy setting.

In addition, nerve-sparing techniques at the time of bilateral RPLND result in rates of antegrade ejaculation similar to those seen with modified templates. Common features of retroperitoneal relapse after RPLND include a predilection for the left para-aortic region and the increased prevalence of teratomatous elements. Both reoperative and, arguably, primary RPLND or PC-RPLND should be performed in tertiary centers by experienced surgeons in an effort to reduce perioperative morbidity and improve oncologic outcomes.
